# An Extraordinary Cause of the Sucking Difficulty: Ecthyma Gangrenosum

**DOI:** 10.1155/2016/8502150

**Published:** 2016-04-06

**Authors:** Nesrin Ceylan, Nihat Demir, Selami Kocaman, Erdal Peker, Oğuz Tuncer

**Affiliations:** ^1^Department of Pediatrics, Yuzuncu Yil University School of Medicine, 65080 Van, Turkey; ^2^Department of Pediatrics, Division of Neonatology, Yuzuncu Yil University School of Medicine, 65080 Van, Turkey

## Abstract

Ecthyma gangrenosum is a cutaneous lesion often associated with pseudomonas aeruginosa bacteremia, even though it may develop without bacteremia and may originate from other bacterial and fungal organisms. Pseudomonas aeruginosa bacteremia or sepsis, which mainly affects immunocompromised patients, frequently occurs in hospitals. This lesion typically occurs on the extremities and gluteal and perineal regions. In this report we present a case of ecthyma gangrenosum in a premature newborn occurring secondary to pseudomonas sepsis causing sucking dysfunction due to tissue loss in the lip, soft palate, and tongue.

## 1. Introduction

Ecthyma gangrenosum (EG) is generally considered a sign of sepsis by pseudomonas aeruginosa (PA), even though it may also be caused by other organisms [1]. The incidence of all PA bacteremia in patients with cutaneous lesions is 1.3–13% [2, 3]. During septicemia, the microorganisms tend to invade the walls of small blood vessels which results in arterial and venous thrombosis and eventual loss of tissue [4]. To our knowledge, no case of ecthyma gangrenosum secondary to pseudomonas sepsis causing sucking dysfunction secondary tissue loss in the lip, soft palate, and tongue has been reported in literature thus far. We wanted to present this important loss of function occurring secondary to ecthyma gangrenosum in light of present available literature.

## 2. Case Report

**Figure 1:**
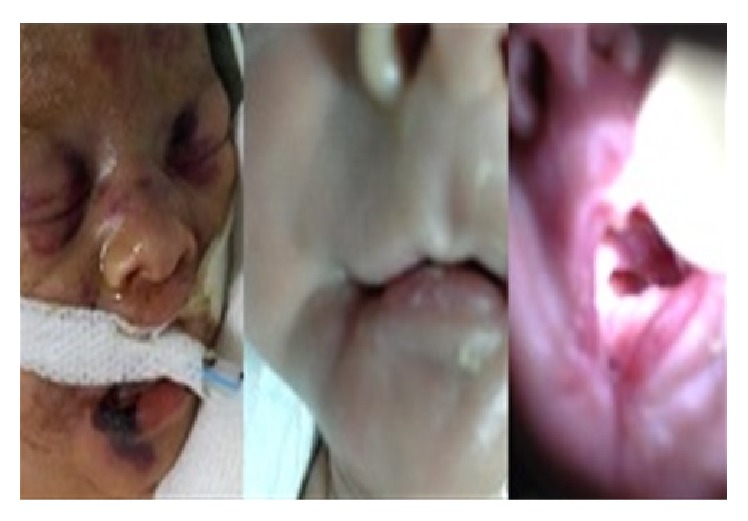
Appearance of necrotic ulcers in the edge of the lip at baseline and one month later.

A male infant was vaginally delivered to a 24-year-old G2P2 mother at 30 weeks of gestation. Apgar scores were 6 and 8 at 1 and 5 minutes, respectively. The infant required respiratory support and was transferred to the neonatal intensive care unit where he was intubated and ventilated. A chest radiograph displayed respiratory distress syndrome and surfactant replacement therapy was given. In accordance with our units' policy, the infant's skin and blood cultures were taken immediately after delivery and the baby was given amoxicillin and gentamicin. At birth, his birth weight was 1450 g (10–25 percentiles), height was 40.5 cm (25–50 percentiles), and his head circumference was 28 cm (25–50 percentiles). On third day of life, these antibiotics were discontinued when the cultures were found to be negative. On 5th day of life, he was seen to clinically deteriorate. Because there was the suspicion of sepsis, he was given vancomycin, amikacin, and topical gentamicin and cultures for blood, urine, and skin lesions were taken and biochemical tests were performed. Biochemical laboratory examinations revealed C-reactive protein (CRP) to be 100 mg/dL (normal range: 0–5 mg/dL), the platelet count was 14 × 10^3^/mm^3^, and the white blood cell count was 9.2 × 10^2^/mm^3^; all other parameters were normal. The next day, rapidly progressive edematous, erythematous, and necrotic plaques with bullae were observed to form on his lips, soft palate, and tongue (Figure 1). Simultaneously, similar lesions developed on his back, gluteal region, and both lower extremities. Three days later, the blood and skin lesion cultures were found to be positive for PA, but the urine culture was negative. According to the results of the culture antibiogram, vancomycin was stopped and ceftazidime was added to the treatment regimen because Gram-negative bacteria are sensitive to ceftazidime, and then the patient started to recover. Five days after the treatment initiation the skin lesions started to improve and on the 10th day of treatment these lesions disappeared; however, due to the loss of tissue in the lips, soft palate, and tongue, the patient developed difficulty in the functions of sucking and swallowing (Figure 1). Because of this dysfunction, the patient was fed with an orogastric tube. He was discharged in good general condition 63 days after birth.

## 3. Discussion

Most cases of ecthyma gangrenosum, which is a rare cutaneous lesion, are associated with pseudomonas aeruginosa bacteremia. A weak mechanical defense barrier and underdeveloped cellular or humoral immunity increased the risk of infection in newborns [4, 5]. In the general population lesions are usually present in the gluteal and perineal regions or on the extremities and trunk, but rarely are they found on the face or neck [6], whereas, in most reported cases of preterm EG, the face is affected as was the case in our patient [7, 8]. Even though literature has shown that most children with EG had either previously undetected immunodeficiencies or transient risk factors such as immunosuppressive therapy that predisposed them to the development of the disease, not many newborn cases have been reported [9]. EG can be confused with other skin lesions in newborns like noma neonatorum and the skin lesions associated with infections such as group A streptococcus,* Aeromonas hydrophila*,* Staphylococcus aureus*,* Serratia marcescens*,* Pseudomonas maltophilia*,* Escherichia coli*,* Candida albicans*, aspergillus species, and mucor species. A lesion resembling EG but caused by* Candida albicans* was reported in a 12-day-old baby [10]. Another rare manifestation of pseudomonas infection in the neonate is noma neonatorum [11]. Even though the diagnosis of EG is mainly clinical, it must be confirmed in blood and skin lesion cultures. If cultures are negative and the diagnosis is still considered biopsy for histopathology and culture should be considered [12]. Treatment is generally aimed at the underlying bacteremia and should include synergistic antibiotics such as an aminoglycoside and an antipseudomonal cephalosporin until the patient stabilizes and the resistance profile of the organism is known. Topical antibiotics can be applied and local debridement can be performed on the lesion [7].

The existence of EG must warn the physician of the potential likelihood of pseudomonal bacteremia, and the early use of appropriate antimicrobial agents is necessary to minimize the high morbidity and mortality associated with pseudomonal infections.

## 4. What Is New?

Despite early diagnosis and treatment of the disease, organ dysfunctions secondary to serious tissue loss in the lesion area can be observed, as was the case in our patient.
